# Exploring the mechanisms of social jet lag in risk decision-making with machine learning models

**DOI:** 10.3389/fpsyt.2026.1872785

**Published:** 2026-09-01

**Authors:** Caixia Wang, Weijie Chen, Nian Xu, Mengxue Xu

**Affiliations:** 1 Educational Development Research Center of Southern Xinjiang, School of Education Science, Kashi University, Kashgar, China; 2 Central China Normal University, Wuhan, China

**Keywords:** behavioral prediction, circadian rhythm, linear regression, machine learning, random forest, risk decision-making, social jet lag, XGBoost

## Abstract

Risk decision-making describes how individuals evaluate uncertain options and accept potential losses in pursuit of rewards. This study used machine learning models to examine whether social jet lag and basic demographic or temporal variables can explain individual differences in risk decision-making. Using 407 valid responses from participants aged 17–23 years, we compared Linear Regression, Random Forest, and XGBoost regression models after grid-search tuning for the ensemble models and 5-fold cross-validation. The models showed limited predictive capability on the held-out test set (Linear Regression: R^2^ = 0.051, RMSE = 7.31; Random Forest: R^2^ = -0.004, RMSE = 7.52; XGBoost: R^2^ = 0.007, RMSE = 7.48). Linear Regression performed slightly better than the two nonlinear ensemble models, indicating that the available variables did not provide meaningful nonlinear predictive gains. Rather than presenting a deployable prediction tool, the study identifies the boundary conditions of using a small set of circadian and demographic features to model risk decision-making, and it highlights the need for richer behavioral, affective, and contextual data in future computational behavioral research.

## 1 Introduction

Social life is often organized around work, school, and other externally imposed schedules. These schedules may conflict with an individual’s biological rhythm, especially among adolescents and young adults who tend to have later sleep timing. Students and working adults may delay sleep because of academic, occupational, or social demands while still needing to wake early the next day. This mismatch can lead to insufficient sleep, delayed sleep onset, daytime sleepiness, and other sleep-related problems (1–4). Social jet lag refers to the discrepancy between biological timing and socially required schedules, and it represents a chronic form of circadian misalignment in everyday life (5).

Stable physiological rhythms support health and daily functioning (6). Social jet lag can disrupt this stability by shifting sleep-wake timing away from the internal circadian clock (7). Prior studies have linked social jet lag or related circadian disruption to daytime sleepiness (8), school-related difficulties (9), menstrual symptoms (10), melatonin dysregulation (11), memory impairment (12), depression and anxiety (13), and effort-based decision-making (14). These findings suggest that social jet lag may influence risk decision-making through several pathways: reduced sleep duration, impaired attention and inhibitory control, altered reward sensitivity, and changes in affective regulation.

Risk decision-making refers to the process by which individuals evaluate uncertain choices involving potential gains and losses and then select a course of action (15, 16). Risk-taking behavior is the observable outcome of this decision process. Previous research has shown that sleep loss and circadian misalignment can impair cognitive control and increase vulnerability to impulsive or risky choices (17–20). However, existing studies have generally examined average group differences or specific laboratory manipulations. Less is known about whether naturally occurring social jet lag can explain individual differences in risk decision-making when combined with demographic and behavioral timing variables.

Traditional studies in this area often rely on correlation, analysis of variance, or linear regression. These methods are valuable for testing theory-driven hypotheses, but they may not fully capture nonlinear relationships or interactions among circadian timing, demographic characteristics, and behavioral outcomes. Machine learning methods provide an exploratory framework for testing whether such variables contain enough information to support individual-level prediction. At the same time, machine learning models can be misleading when the available features are sparse. In that situation, complex algorithms may add computational complexity without producing meaningful predictive gains.

Accordingly, this study examines the relationship between social jet lag and risk decision-making in young adults. The study has two objectives. First, it evaluates whether social jet lag is associated with risk-taking scores measured by the Balloon Analogue Risk Task (BART). Second, it tests whether a limited feature set consisting of social jet lag, age, gender, and test hour can support individual-level prediction using Linear Regression, Random Forest, and XGBoost models. The study is therefore framed as an exploratory assessment of model feasibility and interpretability rather than as a claim that the current models can accurately predict risk decision-making.

## 2 Methods

### 2.1 Participants

During the initial phase, more than 500 response records were collected from eligible young adult participants. Records were screened for missing core variables, invalid age or gender information, unavailable risk-taking scores, unavailable social jet lag values, unavailable test time, and careless completion. The final analytic sample included 407 valid respondents aged 17–23 years (M age = 19.14, SD age = 0.80). Based on the coding scheme used in the dataset (1 = male, 2 = female), the sample included 185 males (45.5%) and 222 females (54.5%). Participants provided informed consent before completing the study procedures. The study involved human participants and was conducted in accordance with the ethical standards of the institutional research ethics committee and the Declaration of Helsinki.

### 2.2 Data sources, data collection and measurement

#### 2.2.1 Participant recruitment and experimental procedure

Participants were recruited through distribution of the study link to eligible young adults. Participation was voluntary. After reading the study information and providing informed consent, participants reported demographic information, completed sleep-schedule items used to calculate social jet lag, and then completed the BART risk decision-making task. The system recorded the time at which the risk task was completed. After data collection, the dataset was screened according to the exclusion rules described above, and the cleaned dataset was used for descriptive analysis, feature extraction, model training, and model evaluation.

#### 2.2.2 Social jet lag measurement

Social jet lag was calculated from sleep schedule information as the absolute difference between the midpoint of sleep on free days and the midpoint of sleep on work or school days. A higher value indicates greater misalignment between an individual’s preferred sleep timing and socially required schedules. The final analysis file contained one social jet lag value for each participant.

(1) SJL=MSF−MSW

where MSF denotes the midpoint of sleep during holidays (weekends or rest days), while MSW represents the midpoint of sleep on workdays (Equation 1) (21). The difference between these two values indicates the degree of discrepancy between an individual’s socially prescribed sleep pattern and their natural sleep cycle. A higher score signifies a greater degree of social jet lag.

#### 2.2.3 Risk decision-making measurement

The Balloon Analogue Risk Task (BART) was used to measure risk decision-making because it captures behavior under uncertainty and has ecological validity in laboratory studies of risky choice (22). In the BART, participants inflate a virtual balloon to earn points. Each additional pump increases the reward but also increases the probability that the balloon will burst. If the balloon bursts, points for that balloon are lost; if the participant stops before the burst point, the accumulated points are saved. The task included 30 balloons. The risk-taking score was calculated as the average number of pumps on unexploded balloons, with higher scores indicating a greater tendency to accept risk under uncertainty.

#### 2.2.4 Other variable measurement

The time at which each participant completed the risk decision-making task was recorded in hours, minutes, and seconds and was converted to an hour-level feature for analysis. Gender was coded as a binary variable (1 = male, 2 = female). Age was treated as a continuous variable ranging from 17 to 23 years.

### 2.3 Data analysis

#### 2.3.1 Data preprocessing

Data preprocessing focused on preserving a transparent analytic sample. Records with missing values in the core variables were excluded before analysis. The final dataset used in the present analysis contained no missing values. The interquartile range (IQR) rule was used to inspect potential outliers in risk-taking scores, as calculated using Equations 2–4. Because unusually high BART scores can represent valid high-risk task performance rather than data entry error, extreme but valid observations were retained and the resulting skewed distribution was reported in the Results section.

(2) IQR=Q3−Q1

(3) Lower Bound=Q1−1.5×IQR

(4) Upper Bound=Q3+1.5×IQR

The risk-taking score distribution was strongly right-skewed. The highest observed score was 56.69, which was above the IQR upper-bound criterion. This value was retained because it represented a possible BART performance pattern rather than an impossible value. Retaining such values is important for risk decision-making research, but it also makes individual-level prediction more difficult.

#### 2.3.2 Feature engineering

Feature engineering was kept deliberately parsimonious to preserve interpretability. The primary reported model used four predictors: age, gender, social jet lag, and the hour at which the risk decision-making task was completed. The original time stamp was reduced to an hour-level feature. Continuous predictors were standardized with z-scores before model training, as calculated using Equations 5. Two exploratory interaction terms were inspected during coding, but they did not improve held-out predictive performance and were not retained in the reported model. This decision addresses the limited feature space and reduces the risk of unnecessary algorithmic complexity.

(5) $$X_{\text{scaled }}=\frac{X-\mu }{\sigma }$$

In the z-score transformation, x denotes the original feature value, mu denotes the feature mean, and sigma denotes the feature standard deviation. Mutual information (MI) was used as an additional exploratory indicator of nonlinear dependence between each feature and the target variable. Higher MI scores indicate that a feature contains more information about the risk-taking score, but MI values alone do not establish causal influence or practical predictive utility.

(6) $$MI(X,Y)=\sum_{x\in X}\sum_{y\in Y}p(x,y)\log(\frac{p(x,y)}{p(x)p(y)})$$

In Equation 6, p(x) and p(y) denote the marginal probability distributions, and p(x,y) denotes the joint probability distribution. MI quantifies statistical dependence between each feature and the target variable. A higher MI score indicates stronger dependence, whereas a low MI score indicates that the feature contains little information for predicting the target variable.

#### 2.3.3 Model selection, training and optimization

- Linear Regression was included as a simple baseline model because the outcome variable was a continuous risk-taking score. Random Forest (RF) and XGBoost were selected as benchmark ensemble learning models because they are commonly used for tabular prediction tasks and can model nonlinear relationships without imposing a strict linear functional form. In this study, the three models were used to test whether the available circadian, demographic, and temporal variables contained enough signal for prediction. The comparison was not used to imply that a complex model is automatically superior to simpler statistical approaches.
- RF constructs a large number of decision trees, each using a random sample of data and features, and makes predictions by averaging all trees’ outputs (regression tasks) or through majority voting (classification tasks). This helps reduce prediction variability and makes the model more robust.
- XGBoost’s concept involves sequentially training decision trees, with each new tree attempting to correct the errors (residuals) of the previous one. This process is iterated multiple times, resulting in a strong model built from weak learners. XGBoost employs advanced techniques such as gradient boosting and regularization to enhance accuracy and prevent overfitting.

The cleaned dataset was split into training and test sets using an 80:20 ratio with a fixed random state of 42. Linear Regression was fitted as an untuned baseline, whereas Random Forest and XGBoost hyperparameters were tuned using GridSearchCV with 5-fold cross-validation on the training set. The final models were evaluated on the held-out test set using MSE, RMSE, MAE, R^2^, and MAPE. Cross-validation RMSE was additionally reported to assess stability across data partitions.

#### 2.3.4 Experimental environment

- Hardware environment: Intel Core i7 processor, 16GB RAM.
- Software environment: Python 3.10, scikit-learn 1.7.1, XGBoost 3.0.2, pandas 2.3.1, numpy 2.2.6.
- Random state: All experiments used a fixed random state of 42 to support reproducibility.

## 3 Results

### 3.1 Descriptive statistics

**Figure 1:**
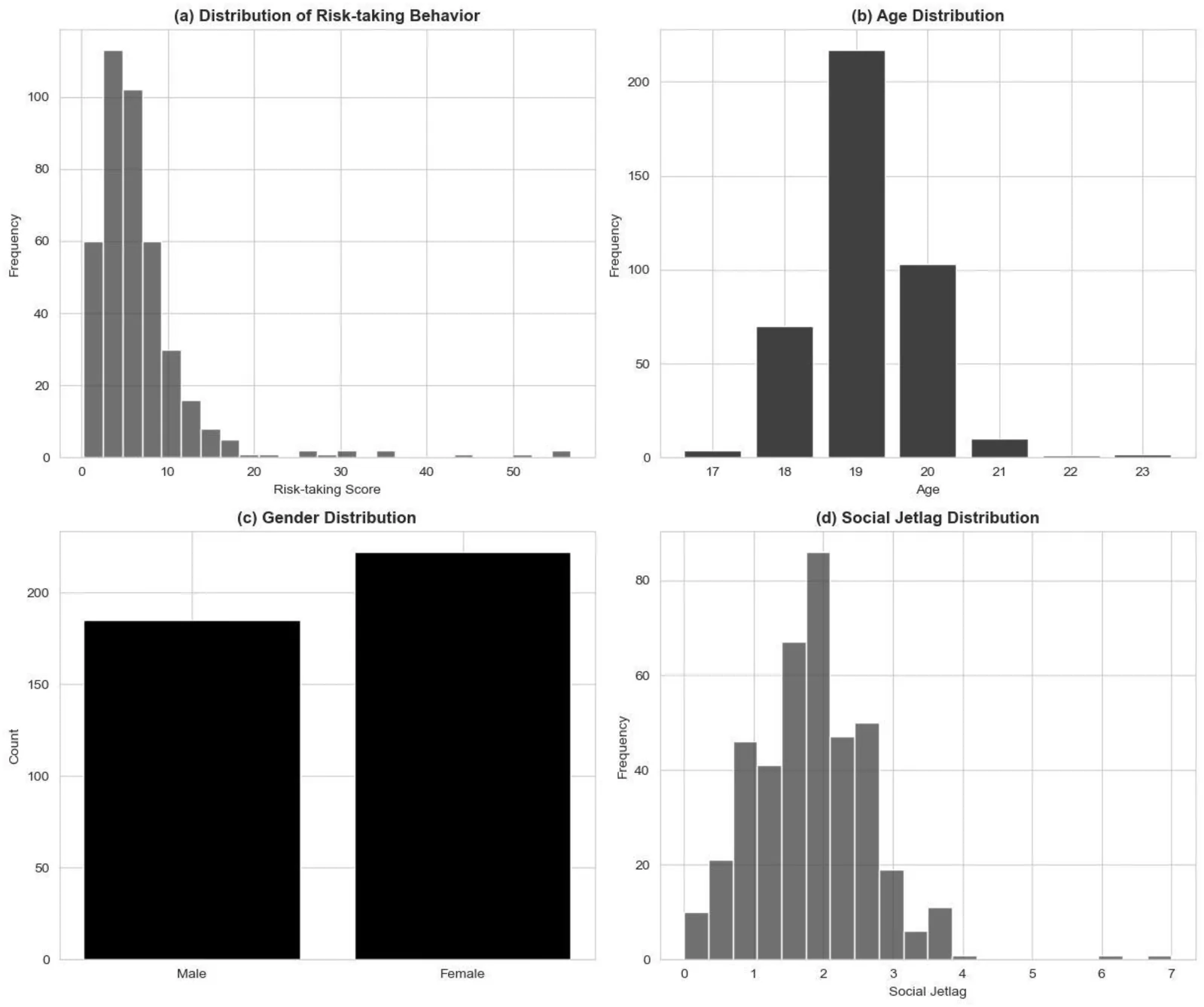
Data distribution chart. **(a)** Distribution of risk-taking score; **(b)** Age distribution; **(c)** Gender distribution; **(d)** Social jetlag distribution.

The descriptive statistics are presented in **Table 1** and **Figure 1**. The risk-taking score showed a pronounced right-skewed distribution, indicating that most participants showed low to moderate risk-taking while a small number showed very high scores. The skewness value was 4.245 and the kurtosis value was 24.423. This non-normal target distribution helps explain why the three models compressed predictions toward the mean and struggled to capture extreme risk-taking scores.

**Table 1:** Basic sample statistics.

Variable	Mean	Standard deviation	Minimum	Maximum	Median
Age	19.14	0.80	17	23	19
Risk score	6.79	6.61	0.24	56.69	5.45
Social jet lag	1.81	0.83	0	7	1.75

The analysis proceeded from descriptive statistics to feature correlation analysis, mutual information analysis, model fitting, cross-validation, and visual inspection of prediction and residual patterns. This sequence was used to evaluate both predictive performance and interpretability.

### 3.2 Feature correlation analysis

**Figure 2:**
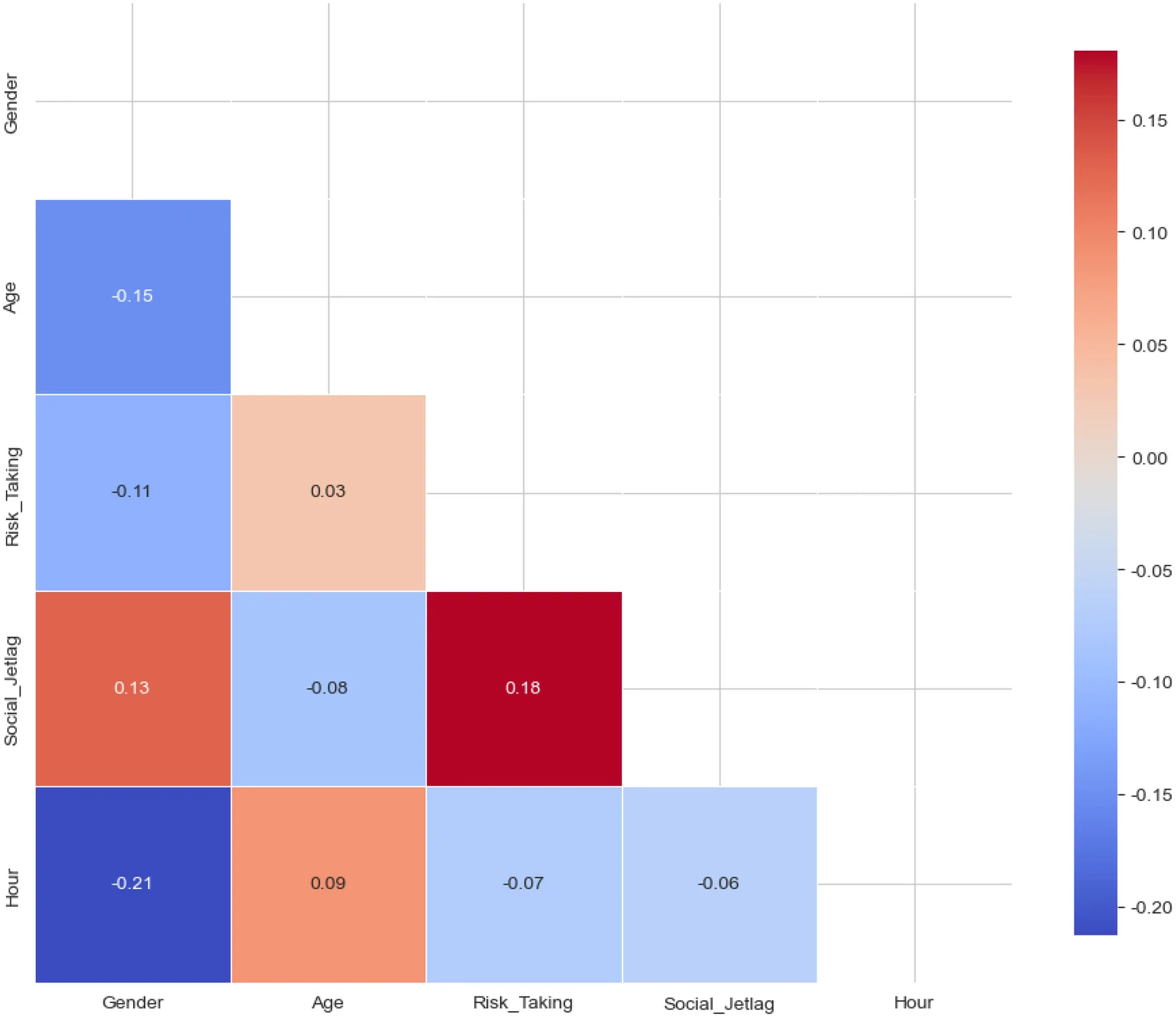
Feature correlation matrix.

As shown in **Figure 2**, the correlation matrix indicates that all observed relationships were weak. Social jet lag had the strongest positive correlation with risk-taking scores (r = 0.18), suggesting that participants with greater social jet lag tended to show slightly higher risk-taking. Gender showed a weak negative correlation with risk-taking scores (r = -0.11), and hour showed a very weak negative correlation (r = -0.07). Age had almost no correlation with the target variable (r = 0.03).

These correlations should be interpreted cautiously. The observed values are too small to support strong claims about individual prediction, but they are consistent with the possibility that circadian misalignment contributes modestly to risk decision-making when combined with other contextual and individual factors.

### 3.3 Feature importance analysis

As presented in **Table 2**, mutual information analysis identified social jet lag as the highest-ranked feature (MI = 0.0263), followed by hour (MI = 0.0231), gender (MI = 0.0164), and age (MI = 0.0033). These values indicate a weak feature signal rather than strong predictive information. Therefore, the MI results support an exploratory association but do not justify claims of accurate individual-level prediction.

**Table 2:** Feature mutual information scores.

Feature	MI score	Ranking
Social jet lag	0.0263	1
Hour	0.0231	2
Gender	0.0164	3
Age	0.0033	4

**Figure 3:**
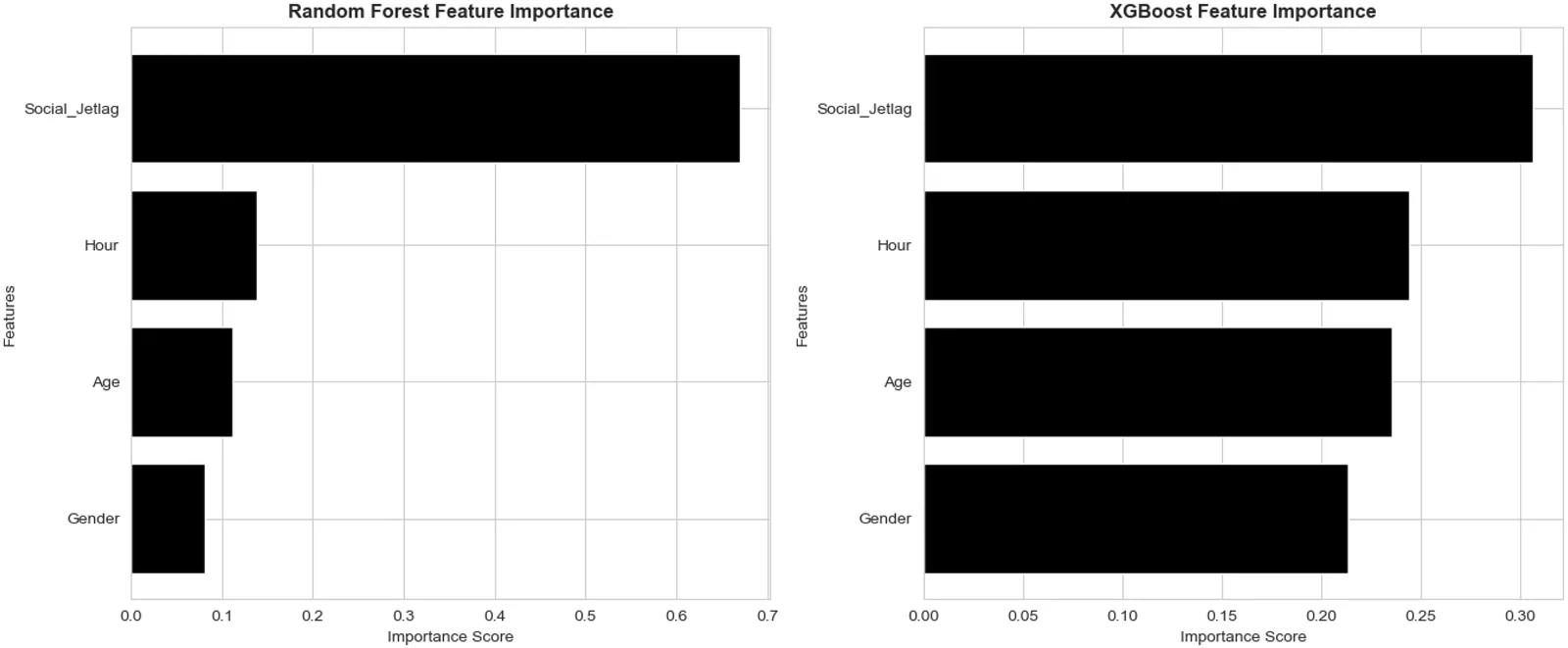
Feature importance comparison.

Model-specific feature importance was then compared for the two nonlinear ensemble algorithms, Random Forest and XGBoost (**Figure 3**). Social jet lag was the most important feature in both ensemble models, but the overall predictive performance remained weak. This pattern suggests that social jet lag is the most informative variable within the present limited feature set, while also confirming that the feature set is insufficient for reliable individual prediction.

### 3.4 Model performance evaluation

Linear Regression was fitted as the baseline model without hyperparameter tuning. Grid search selected 300 trees, max_depth = 10, min_samples_split = 10, and min_samples_leaf = 4 for the Random Forest model. For XGBoost, the selected configuration used 100 estimators, learning_rate = 0.01, max_depth = 3, and subsample = 0.7. These settings were relatively conservative and helped reduce overfitting risk, but they did not overcome the limited predictive signal in the available features. The optimal parameter code example is shown in **Algorithm 1**.

Algorithm 1 **Optimal parameter code example.**

def print_best_parameters(rf_model, xgb_model):   “““Print optimal hyperparameters”““   print(“=== Optimal hyperparameters ===“)   print(“Random Forest optimal hyperparameters:”)   for param, value in rf_model.best_params_.items():      print(f” {param}: {value}”)   print(“\nXGBoost optimal hyperparameters:”)   for param, value in xgb_model.best_params_.items():      print(f” {param}: {value}”)   # Save to filewith open(‘best_parameters.txt’, ‘w’, encoding=‘utf-8’) as f:   f.write(“Random Forest optimal parameters:\n”)   for param, value in rf_model.best_params_.items():      f.write(f”{param}: {value}\n”)   f.write(“\nXGBoost optimal parameters:\n”)   for param, value in xgb_model.best_params_.items():      f.write(f”{param}: {value}\n”)

Test-set performance confirmed that all three models had limited predictive capability. Linear Regression produced R^2^ = 0.051 and RMSE = 7.31, Random Forest produced R^2^ = -0.004 and RMSE = 7.52, and XGBoost produced R^2^ = 0.007 and RMSE = 7.48 (**Table 3**). Linear Regression showed the lowest RMSE and the highest R^2^, but the improvement was small and should not be interpreted as evidence of a practically useful model. The very low R^2^ values indicate that the current feature set explains only a small fraction of individual variation in risk-taking scores.

**Table 3:** Comprehensive comparison of model performance.

Index	Linear regression	Random forest	XGBoost	Interpretation
MSE	53.46	56.57	55.95	Linear Regression lowest
RMSE	7.31	7.52	7.48	Linear Regression lowest
MAE	3.96	4.18	3.80	XGBoost lowest
R2	0.051	-0.004	0.007	All very low
MAPE	77.04%	81.48%	75.60%	XGBoost lowest

To assess model stability and generalization, 5-fold cross-validation was performed on the training data. In each fold, four subsets were used for training and one subset was used for validation. RMSE was calculated for each fold. The results are shown in **Table 4**.

**Table 4:** Cross-validation RMSE results.

Fold	Linear regression	Random forest	XGBoost
1	4.077	4.451	4.494
2	9.686	9.909	9.731
3	4.514	4.923	4.618
4	6.131	6.176	6.159
5	5.046	5.256	5.202
Average	5.891	6.143	6.041
Standard deviation	2.018	1.966	1.937

Linear Regression achieved the lowest average cross-validation RMSE, followed by XGBoost and Random Forest, but the differences were small. The large variation across folds indicates that prediction was sensitive to data partitioning, which is expected for a small and skewed behavioral dataset. The cross-validation results therefore reinforce the conclusion that the present models are exploratory rather than ready for applied screening or individual diagnosis.

**Figure 4:**
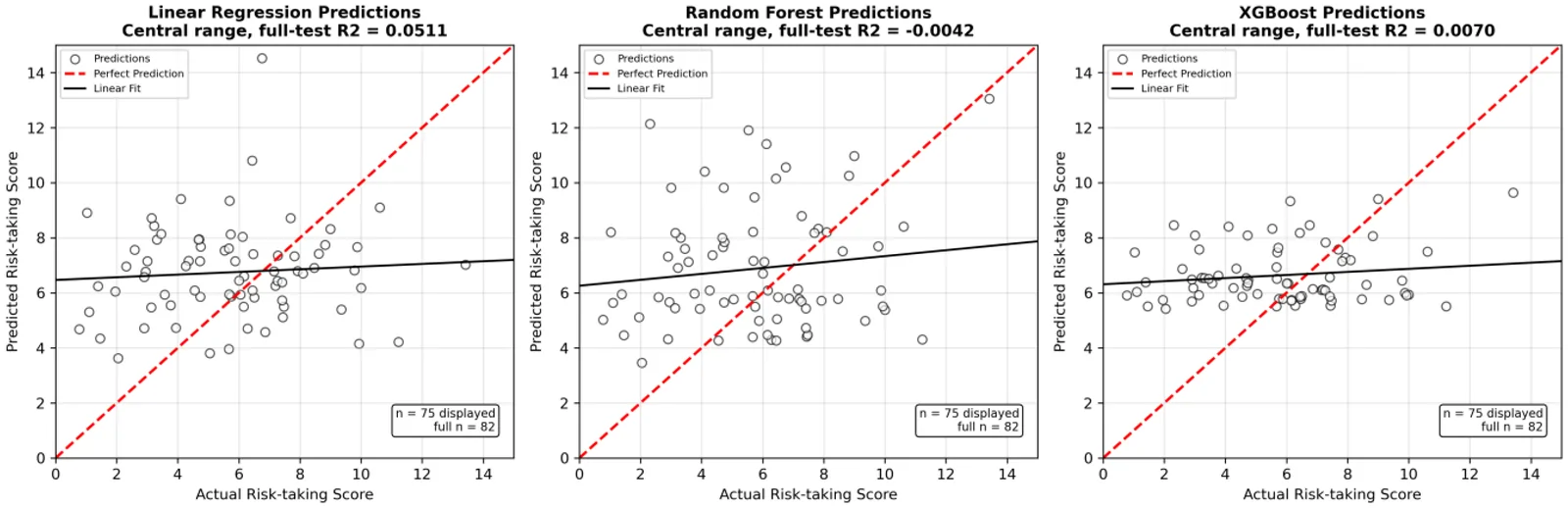
Central-range comparison of actual and predicted values across three models.

Visual inspection of predictions further illustrates the modeling limitation. **Figure 4** presents a zoomed central-range view of the test-set predictions for Linear Regression, Random Forest, and XGBoost (actual risk-taking score <= 14.86, based on the 1.5 IQR upper-bound criterion; n = 75 displayed). The seven high-tail observations were not removed from model evaluation and are omitted only from this visual panel to make the dense prediction pattern readable. In the displayed central range, all three models produced predictions concentrated in a narrow band, indicating limited individual-level discrimination even within the central distribution.

**Figure 5:**
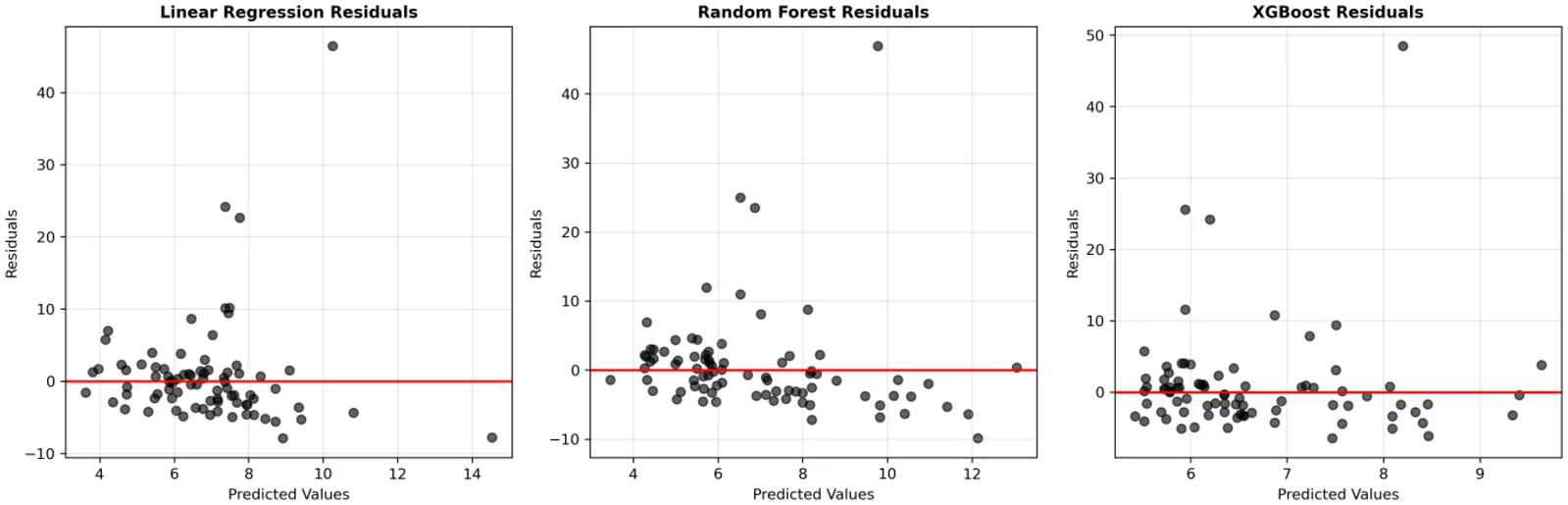
Residual analysis.

The residual plots (**Figure 5**) show that prediction errors were largest for high-risk participants across all three models. The residual standard deviations were 7.32 for Linear Regression, 7.54 for Random Forest, and 7.47 for XGBoost, confirming that the models had difficulty capturing individual differences at the upper end of the risk-taking distribution. The residual pattern indicates systematic underprediction of extreme risk-taking rather than random error alone.

### 3.5 Contextualizing model performance against prior behavioral prediction studies

To contextualize the weak performance observed here, **Table 5** compares the present study with selected behavioral prediction studies that used richer digital or behavioral data sources. Prior studies using large-scale digital traces or broader psychoeducational variables have reported substantially higher predictive performance (23, 24). In contrast, the present study used only four predictors and obtained a best test-set R^2^ of 0.051 from Linear Regression, while the two nonlinear ensemble models remained near zero. The comparison indicates that the low performance is not merely a model-selection issue; it reflects the limited information contained in the available variables for predicting individual risk decision-making.

**Table 5:** Comparative performance of behavioral prediction studies.

Study	Task	Data type	Performance
Kosinski et al. (23)	Predicting personality traits	Facebook activity data	R^2^ = 0.30-0.40
Touloupis et al. (24)	Predicting internet addiction	Behavioral survey data	Accuracy about 75%
In this study	Predicting risk decision-making	Experiment + temporal	Best R^2^ = 0.051 (Linear Regression); XGBoost R^2^ = 0.007

Data richness, sample size, and task complexity likely explain this performance gap. Risk decision-making is influenced by transient affective states, reward sensitivity, inhibitory control, situational context, prior experience, and task-specific learning. The present dataset did not include these variables. Therefore, the main value of the analysis is to show that social jet lag alone, even when combined with age, gender, and test hour, is insufficient for robust prediction.

## 4 Discussion

The central finding of this study is that social jet lag showed a weak association with risk decision-making, but the available variables did not support accurate individual-level prediction. This distinction is important because social jet lag and circadian misalignment have been linked to sleep, affective, cognitive, and decision-related outcomes in prior research (20, 25–27). A variable can be theoretically relevant and still be insufficient for prediction when measured alone or with only a few demographic covariates. The very low R^2^ values therefore do not invalidate the relevance of circadian misalignment; rather, they indicate that risk decision-making is a multidetermined behavior that requires richer measurement of impulsivity, affective state, executive control, and task context (15, 16, 28, 29).

Theoretically, the results support a cautious interpretation of social jet lag as a contextual risk-related factor. Social jet lag may affect decision-making through fatigue, reduced attention, impaired inhibitory control, altered reward sensitivity, or affective dysregulation, which is consistent with evidence linking circadian disruption and sleep loss to attention, inhibition, decision-making, effort-based choice, and reward valuation (14, 30–32). However, the present data cannot identify these mechanisms directly. Future studies should measure sleep duration, chronotype, executive function, mood, stress, reward sensitivity, and situational context to test these pathways more explicitly (28, 29, 32).

Methodologically, the study demonstrates a boundary condition for applying machine learning in behavioral research. Linear Regression performed slightly better than Random Forest and XGBoost, indicating that the available predictors did not provide clear nonlinear gains. Random Forest and XGBoost are capable algorithms, but their performance depends on the information contained in the predictors. Studies using richer digital traces or broader psychoeducational variables have reported stronger prediction, which supports the interpretation that data richness is central to behavioral model performance (23, 24). With only a small set of features and a skewed target distribution, more complex algorithms mainly compressed predictions toward the mean. This finding addresses the concern that complex models may create an impression of sophistication without improving interpretability or predictive value.

Practically, the findings suggest that social jet lag should not be used as a stand-alone indicator for identifying individuals at risk of risky decision-making. At most, it may serve as one component of a broader assessment that includes sleep quality, chronotype, emotional state, impulsivity, executive control, and situational risk exposure, because prior work links sleep and circadian factors to health, affective symptoms, attention, and risk-related behavior across different populations (20, 25–27). The study therefore provides a negative but useful result: observable circadian misalignment alone is not enough for reliable behavioral prediction in this sample.

The study contributes to the literature in three ways. First, it connects social jet lag research with risk decision-making rather than treating sleep timing only as a health or academic-performance variable, extending prior evidence on social jet lag, cognitive control, and risk-related decision processes (25–27, 32). Second, it uses machine learning as a diagnostic tool for testing the predictive sufficiency of available features, not merely as a performance-maximization technique. Third, it reports weak predictive results transparently, which helps define the data requirements for future computational behavioral models and is consistent with the stronger performance observed when richer behavioral or digital records are available (23, 24).

Several limitations remain. The sample was relatively small and homogeneous, with participants aged 17–23 years. The feature set was limited to age, gender, social jet lag, and test hour, and did not include psychological, affective, socioeconomic, or situational variables that may influence risk decision-making. The BART provides an experimentally tractable measure of risk-taking, but it cannot capture all real-world forms of risky decision-making (22). The skewed target distribution and extreme valid scores also made prediction difficult. Finally, the cross-sectional design prevents causal inference about the effect of social jet lag on risk decision-making.

## 5 Conclusion

This study examined whether social jet lag and a small set of demographic and temporal variables could explain individual differences in risk decision-making. Social jet lag showed the strongest association among the available predictors, but Linear Regression, Random Forest, and XGBoost all produced very low predictive performance. Linear Regression performed slightly better than the two nonlinear ensemble models, but the current feature set remains insufficient for reliable individual-level prediction. Future work should use larger and more diverse samples, richer behavioral and psychological features, and theory-guided model comparison to clarify the mechanisms linking circadian misalignment and risk decision-making.

## Data Availability

The raw data supporting the conclusions of this article will be made available by the authors, without undue reservation.
